# Nucleosomes in mammalian sperm: conveying paternal epigenetic inheritance or subject to reprogramming between generations?

**DOI:** 10.1016/j.gde.2023.102034

**Published:** 2023-04

**Authors:** Laura Gaspa-Toneu, Antoine HFM Peters

**Affiliations:** 1 Friedrich Miescher Institute for Biomedical Research, Maulbeerstrasse 66, 4058 Basel, Switzerland; 2 Faculty of Sciences, University of Basel, 4056 Basel, Switzerland

## Abstract

The genome of mammalian sperm is largely packaged by sperm-specific proteins termed protamines. The presence of some residual nucleosomes has, however, emerged as a potential source of paternal epigenetic inheritance between generations. Sperm nucleosomes bear important regulatory histone marks and locate at gene-regulatory regions, functional elements, and intergenic regions. It is unclear whether sperm nucleosomes are retained at specific genomic locations in a deterministic manner or are randomly preserved due to inefficient exchange of histones by protamines. Recent studies indicate heterogeneity in chromatin packaging within sperm populations and an extensive reprogramming of paternal histone marks post fertilization. Obtaining single-sperm nucleosome distributions is fundamental to estimating the potential of sperm-borne nucleosomes in instructing mammalian embryonic development and in the transmission of acquired phenotypes.

**Current Opinion in Genetics & Development** 2023, **79**:102034

This review comes from a themed issue on **Developmental Mechanisms, Patterning and Evolution**

Edited by **Haruhiko Koseki**

For complete overview of the section, please refer to the article collection, “Developmental Mechanisms, Patterning and Evolution (2023)”

https://doi.org/10.1016/j.gde.2023.102034

0959-437X/© 2023 The Author(s). Published by Elsevier Ltd. This is an open access article under the CC BY license (http://creativecommons.org/licenses/by/4.0/).

## Introduction

Nucleosomes constitute the first layer of chromatin organization in eukaryotes. Through their presence, their post-translational modifications (PTMs), and the diversity of histone variants, nucleosomes regulate numerous cellular processes. In mammalian sperm, small basic proteins called protamines (PRMs) are the main constituents of chromatin. PRMs bind directly to DNA inducing circularization into toroidal structures (Box 1) [1]. Nonetheless, early biochemical studies and revisited quantifications estimate residual histones in mature sperm as ~1–2% in mouse and ~4–10% in human compared with somatic genome equivalents 2, 3, 4, 5. Histone PTMs inherited from oocytes serve allele-specific gene-regulatory functions during pre- and postimplantation development [6]. Whether histones residing in sperm are similarly instructive for functions post fertilization remains unclear. Here, we review the following open questions: (i) are certain nucleosomal compositions preferentially retained or exchanged by protamines during spermatogenesis? (ii) Are nucleosomes retained at specific locations within the sperm genome? (iii) To what degree do histone content and distribution vary between spermatozoa? (iv) To what extent does sperm nucleosome-borne information withstand embryonic reprogramming?

Box 1 **Chromatin remodeling during mammalian spermatogenesis.**

The process of sperm generation, called spermatogenesis, initiates in testes at puberty in humans or few days after birth in mice. Initially, spermatogonial precursor germ cells expand through several mitotic divisions. Germ cells then progress as spermatocytes through a lengthy meiotic prophase during which parental genomes exchange genetic information via meiotic recombination. Following a reductional and an equational cell division, the resulting haploid and genetically diversified spermatids remain functionally interconnected due to incomplete cytokinesis. Such round spermatids further develop into mature sperm through a complex cell differentiation process termed spermiogenesis. During spermiogenesis, cellular structures that are required for fertilization develop, such as the flagellum and the acrosome. Further, transcription is globally shut down, the nucleus elongates and condenses, and the cytoplasm is shed. Nuclear compaction co-occurs with extensive displacement of histones from the genome and incorporation of protamines following an apical–caudal directionality. Whereas all mammals express PRM1, primates, most rodents, and some other placental mammals also express PRM2 [7]. PRM2 is translated as a precursor and matures through proteolytic cleavage of its N-terminal sequence. While nucleosomes are directly replaced by protamines in many species, mice, humans, and several other mammals use TNPs (TNP1 and TNP2) as additional regulators of spermatid chromatin remodeling. After the histone-to-protamine exchange, elongating spermatids detach as testicular sperm from the seminiferous epithelium and subsequently passage through the epididymis, a long-convoluted tube (~1 m in mouse and ~4–6 m in humans). Within its three segments, the caput, corpus, and caudal epididymis, compaction of sperm chromatin is further stabilized by intra- and interprotamine disulfide bonds, and sperm acquire fertilization competence and remain stored until ejaculation.

## Nucleosome eviction and retention during the histone-to-protamine exchange

**Figure 1:**
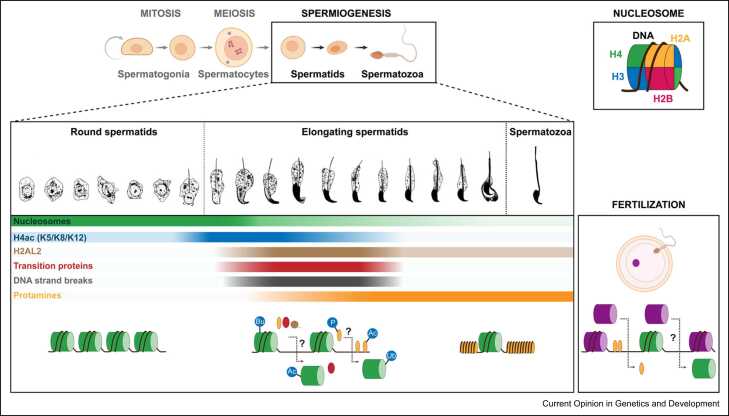
Overview illustrating the exchange of nucleosomes by protamines during mammalian spermiogenesis (Box 1). Nucleosomes consist of 147 bp of DNA wrapped around a histone octamer core containing two copies of the core histones H2A, H2B, H3, and H4. Beyond H4, variants of each core histone incorporate into the genome during meiosis and spermiogenesis. The H2AL2 variant interacts with and contributes to the deposition of TNPs and/or PRMs into chromatin. Several histone PTMs are upregulated at the onset of spermatid elongation. H4 acetylation (H4ac) and potentially other PTMs facilitate histone displacement partly by engaging nucleosome-removing machineries. Conversely, histone PTMs such as butyrylation (Bu) repel certain of such activities. PTMs on TNPs and PRMs regulate incorporation in and removal of these proteins from chromatin, as well as compaction of chromatin. Detailed understanding on the actual mechanisms of histone displacement, retention, and TNP/PRM loading is largely missing. Similarly, the extent by which paternal nucleosomes are exchanged by maternal histones at fertilization remains unclear. Ac: acetylation, P: phosphorylation, Ub: ubiquitination.

The replacement of histones by protamines initiates in early elongating spermatids upon translation of transition proteins (TNPs) and PRMs (Box 1) (Figure 1). While TNP1 and TNP2 are not required for histone eviction, they are necessary for proper sperm chromatin condensation, PRM2 proteolytic cleavage, and proficient fertility 1, 7. PRM1 and PRM2 are instead essential and nonredundant for histone displacement, as well as for sperm chromatin compaction, DNA integrity, and male fertility in mice and humans 1, 7. Before histone eviction, numerous testis-specific histone variants load onto chromatin and multiple histone PTMs become upregulated in early elongating spermatids. Such changes are thought to collectively increase chromatin accessibility, thereby facilitating nucleosome displacement by protamines [8]. Despite the molecular mechanisms driving the histone-to-PRM exchange remain poorly understood, potential roles of certain histone PTMs and variants in favoring histone retention or eviction are emerging.

*Roles of histone PTMs:* Mounting evidence indicates that histone H4 acetylated at N-terminal lysine (K) residues (H4ac) is targeted for removal. While predominantly located at transcriptionally active promoters in round spermatids [9], H4ac levels are globally upregulated upon spermatid elongation 10, 11, 12. Subsequently, H4ac dramatically decreases concomitantly with histone eviction, resulting in only ~2–5% of H4 peptides acetylated at residues K5, K8, or K12 in mature sperm [13]. *In vitro,* protamines displace hyperacetylated nucleosomes more efficiently than unmodified ones [14]. Preventing H4ac upregulation during spermatid elongation halts chromatin remodeling, spermatid development, and frequently leads to increased histone content in late spermatids and/or sperm 15, 16. Two mechanisms have been proposed to mediate acetylated-dependent removal of histones in spermatids: (i) a specialized proteasome that binds hyperacetylated histones and promotes their timely degradation [17], (ii) a testis-specific acetylation reader, BRDT, which preferentially binds diacetylated H4K5ac/K8ac and presumably mediates histone displacement 18, 19, 20. Binding of BRDT is sterically incompatible when H4K5 is butyrylated (H4K5bu) [9]. In line, nucleosomes bearing H4K5bu resist degradation during spermatid elongation and are either later removed via acetylation-independent mechanisms and/or partly retained in sperm [21]. Histones other than H4 are also modified with Kac, Kbu, and other acylations upregulated in spermatids 22, 23. While some, such as H3K9ac [24] or histone crotonylation [25], correlate with altered nucleosome eviction or PRM incorporation in sperm, more mechanistic research is needed to determine potential contributions of histone PTMs in histone removal.

Defective histone displacement also occurs upon dysfunction of certain DNA damage repair factors and associated PTMs such as poly(ADP-ribosyl)ation (PARylation) and serine (S)- 139 phosphorylation at histone variant H2AX (γH2AX) 26, 27, 28, 29, 30. Both PTMs are upregulated during spermatid chromatin remodeling to repair physiologically induced DNA strand breaks (DSBs). In somatic cells, PARylation of histones directly contributes to their immediate removal up to several kilobases away from DSBs [31]. Histone displacement mechanisms associated to DNA repair therefore likely contribute to spermatid histone eviction, with yet- unexplored effects on nucleosome composition [32].

Interestingly, several histone PTM pathways crosstalk during spermatogenesis. For example, decreased histone ubiquitination upon *Rnf8* deletion co-occurs with reduced H4ac due to diminished chromatin binding of the histone acetyltransferase (HAT) MOF [29]. In turn, downregulation of H4ac in fly spermatids leads to decreased H3K79me3 levels [33]. In contrast, catalytic inactivation of PHF7, a novel E3 ligase for histone H2AK119 and H3K14, downregulates BRDT expression, presumably causing persistence of H4Ac and reducing nucleosome eviction from sperm 34, 35. Disentangling the contribution of individual PTM pathways in spermatid histone removal remains a challenging task.

Contrary to histone displacement, little is known about potential molecular machineries safeguarding nucleosomes from protamine exchange. Proteomics data indicate that most H3K27, H4K20, and H3K9 residues are modified in mature mouse sperm [13]. Particularly, the facultative and canonical repressive marks H3K27me3, H4K20me3, and H3K9me3 resist more prominently protamine replacement through yet-unexplored mechanisms [12].

*Roles of histone variants:* PRMs are thought to bind DNA through arginine-mediated electrostatic interactions causing displacement of DNA-binding factors [36]. PRM1 expression in fibroblasts is sufficient to remove histones from chromatin and induces spermatid-like chromatin condensation, despite testis-specific factors such as BRDT being absent [37]. Nonetheless, growing evidence implicates histone variants in incorporating TNPs and PRMs into chromatin. In the absence of the H2AL2 variant, TNPs mislocalize at perinuclear regions and mutant spermatozoa phenocopy defects of TNP deficiencies [38]. In turn, sperm H2AL2 levels are severely reduced upon expression of PRM2 lacking its N-terminally cleaved fragment, which concomitantly results in decreased protamination [39]. Loading of TNPs and PRMs to DNA might therefore be linked to codeposition and/or physical interaction with histone variants. Indeed, H2AL2 interacts with TNP2 in elongating spermatids and TNPs bind PRM2 in vitro 38••, 39. Interestingly, H2AL2 preferential location at chromocenters is dependent on the H2A.B variant 10, 40, 41. In turn, *H2al2-*deficient sperm contain less variant TH2B, H2AL2’s predominant dimerization partner [38]. These results highlight an intricate functional relationship between histone variants in mediating spermatid chromatin remodeling, possibly in region-specific manners.

*Roles of TNP/PRM PTMs*: PTMs of PRMs and TNPs are emerging as modulators of chromatin remodeling. To modulate their DNA-binding affinity, PRMs become phosphorylated shortly after synthesis and partially dephosphorylated upon loading onto DNA. Dephosphorylation of mouse PRM2 at S56 is required for sperm head morphogenesis and male fertility [42]. Conversely, PRM1 removal following fertilization requires phosphorylation at PRM1 S9 and S43, without which zygotic progression halts [43]. Likewise, preventing acetylation and/or methylation of the rodent-conserved K49 of PRM1 by substitution to alanine alters PRM1 DNA binding and condensation in vitro and perturbs preimplantation development [44]. PRM1^K49A^ sperm contain excessive amounts of histones but lower H4ac, arguing for independent removal of H4ac histones and/or a role of PRM PTMs in fine-tuning replacement of nucleosomes with diverse compositions. Importantly, dysfunction of acetylation-controlling factors frequently leads to TNP/PRM mislocalization. For instance, deletion of the *Sirt1* histone deacetylase (HDAC) or the testis-specific nuclear protein in testis (*Nut*) results in decreased H4ac, elevated histone levels, and impaired TNP/PRM nuclear distribution 16, 45. Acetylation pathways might therefore directly control TNP/PRM targeting to chromatin, which in turn may be required for subsequent histone eviction. In support, p300/CBT acetylates TNP2 in vitro [46] and NUT interacts with p300/CBT, TNP2, and H2AL2, suggesting a potential complex necessary for TNP/PRM incorporation into chromatin [16]. Preventing BRDT nuclear localization also renders TNPs/PRMs mislocalized as in *Nut*, *Sirt1*, and *H2AL2* deficiencies [20], further reinforcing potential direct interactions. Although a direct role of TNP/PRM acetylation in TNP/PRM targeting awaits investigation, the results above highlight an intricate interplay between histone variants, histone PTMs, and TNP/PRM PTMs in the eviction of histones during spermiogenesis.

## Genomic distribution of nucleosomes in mammalian sperm

**Figure 2:**
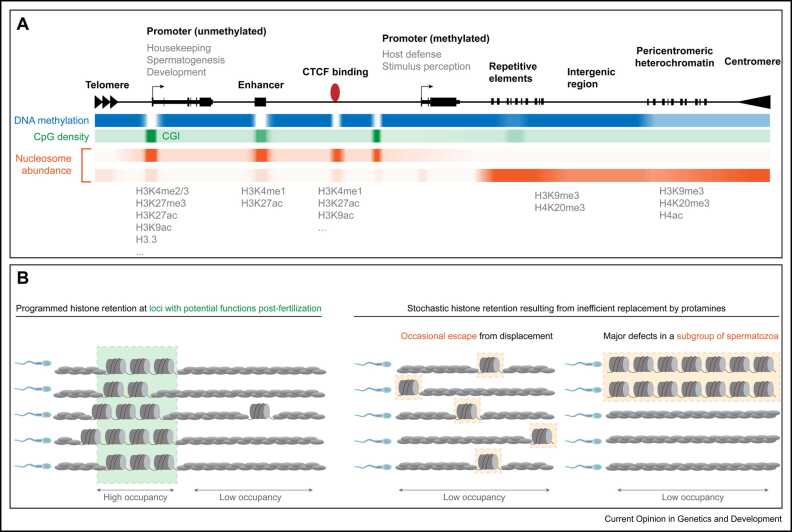
Retention of nucleosomes within the mammalian sperm genome. **(a)** Overview of nucleosome distribution and composition within the mammalian sperm genome. Sperm DNA methylation (blue), CpG density (green), and nucleosome enrichments (orange) are displayed at representative types of genomic loci (black). Current data on nucleosome enrichments represent relative nucleosome abundances in bulk sperm populations. Sperm histone distributions differ between studies, leading to two main models: high abundance of nucleosomes at promoter and distal gene-regulatory regions predominantly lacking DNA methylation and overlapping CpG-rich sequences (upper orange) or preferential retention at intergenic regions and repetitive elements (lower orange). Absolute nucleosome enrichment maps obtained with orthogonal methodologies will help to understand whether specific genomic loci are preferentially packaged by nucleosomes or protamines in sperm. **(b)** Potential modes of heterogeneity in nucleosome retention between individual spermatozoa. Left: nucleosomes or protamines could package defined regions of the sperm genome in a homogeneous mode in most individual spermatozoa (rows) within a population. This would reflect a finely controlled and deterministic exchange of nucleosomes by protamines. Right: inefficiencies in histone replacement could instead render some nucleosomes randomly distributed within the genome of spermatozoa (middle) or lead to severe defects in histone displacement in a fraction of spermatozoa (right).

Three decades ago, biochemical studies first suggested that certain regions of the sperm genome are predominantly packaged with histones, protamines, or both 47, 48, 49. Since then, epigenomic assays revealed nucleosomes at various genomic loci in sperm. Yet, the relative abundances of nucleosomes along genomic regions vary dramatically between studies: initial reports observed nucleosomes predominantly at gene-regulatory regions devoid of DNA methylation (DNAme) 3, 4, 50, 51, whereas subsequent studies proposed a preferential retention at intergenic loci and repetitive elements 52, 53 (Figure 2a). At the core of these discrepancies are technical biases inherent to MNase-seq and ChIP-seq methodologies, which are aggravated by sperm’s unique nuclear architecture (Box 2) [52].

Box 2 **Technical complexity of studying sperm chromatin.**

Protamines compact sperm chromatin several-fold, thereby safeguarding sperm DNA against damaging agents. Hence, studying sperm chromatin frequently requires experimental decondensation of sperm nuclei to grant accessibility to enzymes, antibodies, and other reagents. Pretreatments include permeabilization of membranes, reducing protamine disulfide bonds (e.g., with DTT), and removing PRMs (e.g., by detergents such as NP-40 and sodium deoxycholate [125] or by protamine chaperones [55]). Sperm nuclear pretreatments affect differentially the stability of nucleosomes with specific histone compositions. For instance, histone variants H2AL1/2, TH2A, or H2BL1 detach more readily from spermatid and sperm DNA than their canonical counterparts 10, 126. Hence, strong detergent-based decondensation buffers partially disrupt their binding to DNA, while milder compositions do not allow full solubilization of canonical or other variants such as H2AX after chromatin fragmentation [127]. Similarly, sperm chromatin with repressive H3K27me3 or H3K9me3 PTMs has overall lower solubility than chromatin bearing active H3K4me3 or H3K79me3 [55]. Hence, it is critical to ensure that sperm pretreatments enable complete solubilization of all nucleosome compositions while preventing extraction of histones from less-stable nucleosomes. *In vitro* removal of protamines with Nucleoplasmin2 (NPM2, a protein expressed in oocytes, which removes sperm protamines upon fertilization) greatly aids in granting homogeneous chromatin accessibility [55]. Nonetheless, NPM2 and particularly heparin, a negatively charged glycosaminoglycan frequently used to decondense sperm for immunofluorescence studies, induce histone displacement from DNA under certain conditions 128, 129. Their use requires extensive assessment of potential dose-dependent side effects impacting histone stability.

Chromatin fragmentation methods also dramatically contribute to variations in sperm and somatic cell nucleosome distributions. MNase digests AT-rich DNA faster than GC-rich DNA. Hence, nucleosomes at GC-rich sequences are under-represented in partial MNase digestions, whereas those at AT-rich DNA and with sensitivity to MNase action (i.e., “fragile” nucleosomes) are depleted in complete digestions [130]. Increasing the extent of MNase chromatin digestion shifts nucleosome abundance at GC-rich promoters from under- to over-representation [52]. Moreover, accessible chromatin is also more readily digested than inaccessible. Hence, the physical location of DNA within sperm nuclei may increase technical variability, with peripheral loci such as telomeres and certain gene-rich regions more readily digested than those buried deeper inside the nucleus such as centromeres 67, 131. ChIP-seq nucleosome distributions varied depending on histone antigens examined (e.g., total H3 vs. total H4) [55].

Finally, given the large differences in chromatin composition and organization between sperm and somatic cells, it is crucial to study pure sperm samples. Contamination of somatic or germ precursor cells, combined with partial solubilization of sperm chromatin, may lead to inconclusive results. Accordingly, a recent study has challenged chromatin accessibility, nucleosome and PTM occupancies, and nuclear organization studies published to date arguing that reported results originate from contaminating cell-free DNA present in sperm samples [132].

*Gene promoters:* Numerous studies using diverse technical procedures report nucleosomes at certain classes of gene promoters characterized by overlapping DNA hypomethylated CpG islands (CGIs) 3, 4, 5, 50, 51, 54••, 55••, 56. Many serve housekeeping functions, are commonly expressed in embryonic, somatic, and precursor germ cells, and are similarly modified with active histone marks in sperm (e.g., H3K4me2/3, H3K9ac, and H3K27ac) 4, 5. Likewise, certain highly expressed spermatogenic genes, DNA hypomethylated in sperm but not in soma, retain broad domains of active PTMs. Promoters bearing active histone marks are accessible by ATAC-seq and enriched for RNAPII in sperm despite being transcriptionally inactive 5, 57. Notably, promoter accessibility was not recapitulated in nucleosome occupancy and methylome sequencing (NOMe-seq) experiments in mouse nor human sperm 58, 59, underscoring potential technical variations (Box 2) and/or reflecting differential readouts such as relative enrichment versus absolute population measurements.

Genes involved in embryogenesis, morphogenesis, and cell fate commitment, including developmental miRNAs and noncoding RNAs, retain H3K27me3-marked nucleosomes at their DNA hypomethylated promoters and are occasionally comarked with H3K4me3 3, 4, 5. Such genes are transcriptionally repressed and similarly modified during gametogenesis and embryogenesis. By contrast, non-CGI promoters of tissue-specific genes related to stimulus perception and host defense contain proportionally lower levels of nucleosomes and are marked by DNAme [51]. Hence, nucleosome occupancy and DNAme anticorrelate in sperm chromatin, yet whether DNAme prevents nucleosome retention awaits functional analysis. Transcriptional activity in post-replicative spermatocytes and spermatids also modulates histone variant composition at promoters in sperm. H3K4me3-bearing promoters of previously active genes are highly enriched for H3.3 in sperm, whereas those repressed by H3K27me3 preserve canonical H3 presumably due to reduced transcription-coupled nucleosomal turnover during spermatogenesis [51].

*Distal-regulatory regions, enhancers, CTCF, and TADs:* Several groups reported chromatin accessibility at intergenic regions in mouse and human sperm by ATAC-seq 5, 24, 57. Distal accessible sites (DAS) were shown to be bound by multiple transcription factors (TFs), including CTCF and the pioneer factor FOXA1, suggesting potential regulatory roles [57]. CTCF engages chromatin at several thousand loci in mouse sperm but is absent in human spermatozoa 5, 60. Nucleosomes marked with active PTMs flank CTCF- and FOXA1-bound sites. Such TFs may promote local retention of histones 5, 52, 61, a notion to be further explored mechanistically. Nucleosomes at several hundred DAS contain classic enhancer histone PTMs — H3K4me1 and H3K27ac — suggesting enhancer marking in mouse sperm [5]. Enhancers are short cis-regulatory elements that promote transcription when brought into spatial proximity to their target genes through chromatin looping by CTCF and cohesin complexes. As in soma, both factors organize mouse sperm chromatin into topologically associated domains (TADs) with striking overall similarity to somatic cell chromatin architecture 5, 62. Given that protamines are thought to package sperm DNA into toroidal structures, the presence of soma-like TADs is surprising and was experimentally challenged, revealing the presence of sperm-specific compartments but absence of TADs [63]. While TADs have also been detected in rhesus monkey sperm [64], TADs have not been detected in human spermatozoa [60]. Hence, further research using orthogonal genome architecture mapping approaches is needed to examine why chromosome folding organization in sperm appears to differ between studies and mammalian species [65].

*Centromeres:* Centromeres are chromosomal regions enabling correct chromosome segregation during cell division. They are composed of tandem repetitive DNA (i.e., satellites) and are epigenetically specified by the H3 variant CENP-A. CENP-A locates to centromeres in spermatozoa of human, mouse, and bull, but also of *Xenopus laevis* and *Drosophila melanogaster* 66, 67, 68, 69, 70•. Contrary to most histones, CENP-A levels in bull sperm are comparable to somatic equivalents 68, 71 and most human spermatozoa contain CENP-A by immunostaining [72]. Nonetheless, whether CENP-A quantitatively evades protamine replacement in mammals remains to be ascertained. Chromatin-bound CENP-A has strikingly slow turnover rates in cycling somatic cells [73]. In line, centromere association of CENP-A is stable for over one year in mouse oocytes [74]. In soma, CENP-A stability is favored by intrinsic structural features, interaction with several (non-)centromeric proteins, and *cis-*binding of satellite transcripts, among others [75]. Similar mechanisms may ensure CENP-A retention during oogenesis and spermiogenesis. In *Drosophila*, depletion of CID, the CENP-A ortholog, results in failure to propagate paternal centromeres and to segregate paternal chromosomes during the first embryonic division [70]. Inter- and trans-generational inheritance of neocentromere positioning through men 76, 77 could lend support to paternal CENP-A transmission.

*Pericentromeric heterochromatin (PCH):* centromeres are flanked by pericentromeric heterochromatic domains composed of tandem satellites (i.e. major satellites in mouse), other non-satellite repeats and abundant constitutive heterochromatic PTMs such as H3K9me3 and H4K20me3. Similarly marked nucleosomes are present at PCH in human sperm and are transmitted to the zygote to propagate constitutive heterochromatin during embryogenesis 3, 78. In mouse, recent WB and proteomic studies reported H3K9me3 presence in sperm 12, 55••. Immunofluorescence and ChIP-seq studies reported variable results ranging from loss of H3K9me3 during spermatid elongation to presence of H3K9me3 signal at PCH in sperm 10, 55••, 79. Nucleosome-sized fragments obtained by partial MNase digestion of sperm DNA hybridize to PCH arguing for local nucleosomal presence [80]. Surprisingly, H4ac co-exists with H3K9me3 at PCH in mouse and human sperm and major satellite transcripts remain bound in cis 10, 41, 55••, 78, 79. Contrary to somatic cells, centromeric and pericentromeric DNA is hypomethylated in mouse and human sperm, as in oocytes and pre-implantation embryos 81, 82. In mouse (but not human) zygotes, paternal PCH lacks H3K9me3 immunofluorescence signals, which in turn enables the establishment of alternative repressive heterochromatin by zygotic deposition of H3K27me3 and PRC1 recruitment, until canonical heterochromatin pathways take over in 8-cell embryos 83, 84. Hence, faithful paternal transmission of H3K9me3 at PCH is not required for forming repressive heterochromatin at paternal PCH in early mouse embryos.

*Telomeres and interspersed repetitive elements:* mammalian telomeres are composed of (TTAGGG)n tandem repeats which locate at chromosome ends and preserve the genetic material during DNA replication. In human sperm, telomeres partly release nucleosome ladders upon mild MNase digestions [85]. These results align with salt extraction studies showing that at least a fraction of sperm telomeric DNA contains nucleosomes [49].

Interspersed repetitive elements constitute major parts of mammalian genomes and have the capacity to transpose across the genome. Initial genomic studies reported nucleosome enrichments at certain repeat families in human and bull sperm [53]. Yet, these enrichments were latter attributed to incorrect quantification of so-called ‘multi-mapping sequencing reads’ that align to multiple locations of the genome [86]. Recently, H4K20me3 in human sperm was over-represented at intergenic regions overlapping long interspersed elements, endogenous retroviral sequences, low complexity and simple repeats [87]. Short interspersed elements were instead under-represented, potentially aligning with H3K4me3 enrichment [88]. Due to their high abundance in the genome and their association with distinct classes of genes [89], it is likely that packaging of repeats in sperm highly depends on their type and genomic context.

*Disclaimer:* despite nucleosomes have been localized at loci described above, their relative abundances in sperm vary between studies. Hence, it remains unclear to what extent and in what locations nucleosomes are preferentially retained within mammalian sperm genomes (see also Box 2).

## Variability of histone content and distribution between spermatozoa

If paternally inherited nucleosomes would execute gene regulatory functions after fertilization, one would envisage high nucleosome occupancy at certain loci in all spermatozoa (Figure 2b, left). To date, most genomic data represent only relative nucleosome enrichments within a sperm population. It is thus unclear to what extent genomic histone distributions vary between individual spermatozoa. Remarkably, levels of several H3 and H4 PTMs are similar between sperm of normospermic men, as measured by proteomics [12]. Conversely, histone and PTM immunofluorescence levels differ greatly among spermatozoa of individuals 90, 91, 92. Such heterogeneity in staining could reflect biological variability or result from technical variation induced by decondensation of sperm (Box 2). Calibrated ChIP-seq further supports biological variability in histone marking between loci and hence among spermatozoa. For example, most loci marked by H3K4me3 or H3K27me3 contained proportionally low or moderate levels of the PTMs in sperm populations whereas only a small percentage of loci retained high levels of modified nucleosomes [93]. While some global heterogeneity in histone modifications might originate from precursor germ cell stages, unevenness in nucleosome replacement during spermiogenesis could introduce further variability (Figure 2b, right). A small fraction of nucleosomes could stochastically evade protamine displacement and locate randomly within sperm chromatin. Alternatively, overall protamination could be defective in some spermatozoa. In support, only ~20–35% of sperm stain positively for TH2B in human donors [72]. TH2B-containing spermatozoa are morphologically indistinguishable from TH2B negative yet decondense at faster rates [94], suggesting differential chromatin packaging in subgroups of sperm. Alike, most histone signal quantified by WB and ChIP-seq in mouse caudal sperm originates from only ~10% of spermatozoa [54]. The percentage of sperm with high histone abundance decreases during epididymal transit, arguing for chromatin maturation and/or selective sperm removal within the epididymis [13]. Sperm with presumably “immature” chromatin were less dense and had higher DNA stainability levels (HDS) measured by the Sperm Chromatin Structure Assay (SCSA) [54]. Fertile and infertile men also contain a fraction of HDS spermatozoa. Interestingly, men with increased HDS have higher H3K4me2 levels than individuals with low HDS [95]. Similarly, sperm selected for low motility/density contain more cells positive for HDS, nucleosomes and histone PTMs 90, 96, 97. These results suggest that most spermatozoa of fertile individuals contain considerably smaller amounts of histones than previously thought. The genomic location of nucleosomes reported in prior studies could therefore represent that of a minority of spermatozoa. Hence, there is an urgent need to apply methodologies with single-cell and/or absolute nucleosome occupancy readouts to understand the variability in histone content and genomic distribution between spermatozoa.

## Reprogramming of paternal nucleosomes and functions post-fertilization

Upon fertilization, PRMs are rapidly displaced from paternal chromatin [43]. Maternally provided histones repackage the paternal genome within one-hour post-fertilization in mouse [98]. Whether sperm transmitted nucleosomes are also replaced during this process is uncertain. By immunofluorescence, presumed sperm-borne replication-dependent histones are detected at paternal chromatin at least until zygotic S-phase, after which parental origin is no longer discernible [99]. Sperm-borne H3.3 is instead exchanged by maternal H3.3 within 1–2 h post-fertilization, suggesting a quick reprogramming of sperm H3.3 100, 101, 102. Genomic analyses of histone PTMs in the paternal genome of pre-implantation embryos indicate extensive remodeling of H3K4me3, H3K9me3, H3K27me3 and H3K36me3 103, 104, 105, 106. Though remodeling of paternal H3K4me3 is disputed [107], these results point towards rather restricted inheritance potential of sperm-borne histone PTMs.

Nonetheless, alterations in abundance and genomic distributions of nucleosomes and histone PTMs in sperm frequently associate with poor reproductive outcomes 91, 92, 108. For instance, sperm of asthenoteratozoospermic patients contained reduced levels of H3K9me2/me3, H4K20me2 and H4ac while H3K27/H3K36/H3K79 methylations were equivalent to those of normospermic men [109]. While such chromatin abnormalities could negatively impinge on fertility, mechanistic insights are missing. Intriguingly, mouse embryos generated by injecting nuclei of round spermatids into oocytes (ROSI) have lower developmental rates than those injected with mature sperm nuclei (ICSI) [110]. ROSI-derived zygotes fail to demethylate paternal DNA and abnormally stain with H3K9me2/3 at paternal PCH 111, 112, 113••. Moreover, sustained repression of certain genes in ROSI-derived 2-cell embryos correlates with higher promoter H3K27me3 in round spermatids compared to sperm [113]. This finding possibly argues for round spermatid-derived H3K27me3 suppressing embryonic transcription and preventing the recruitment of epigenetic machineries normally reprogramming the paternal genome. Hence, nucleosomal removal during mammalian spermiogenesis could fine-tune sperm’s capacity to support embryogenesis. In *Xenopus*, however, given the extensive transmission of H3/H4 histones through sperm, loss of H3K4me3 during spermatid remodeling likely ensures transcriptional repression in embryos [114].

Alterations in sperm histone content, composition and distribution have been reported upon exposures of males to chemicals such as vinclozolin, DTT or ethanol as well as to dietary factors including low-protein or folate-deficient diets 107, 115, 116. For example, males fed with low protein diet contain proportionally lower levels of H3K9me2 in sperm [117]. Yet, associations of histone perturbations in sperm to phenotype transmission or altered embryonic expression are generally correlative rather than causal. Overexpression of the H3K4me2 demethylase KDM1A/LSD1 during spermatogenesis leads to trans-generational transmission of developmental defects. While observed reductions in promoter-associated H3K4me2 in sperm was excluded as possible mediator of transgenerational inheritance 118•, 119, increased H3K4me3 levels was proposed to convey transgenerational inheritance 118•, 119. Alike, ~30% of loci with altered H3K4me3 in sperm from mice fed with folate-deficient diet displayed abnormal H3K4me3 levels in 8-cell progeny [107]. Even so, mechanistic evidence of H3K4me3 or other sperm PTMs as vehicles of paternal phenotype inheritance is currently lacking. In addition, environmental perturbations of sperm histone content frequently co-occur with alterations of sperm DNAme and non-coding RNAs composition 118•, 120. Such alterations and more recently sperm chromatin-bound TFs have been mechanistically implicated as means of paternal inheritance 121, 122, 123, 124. Hence, multiple pathways may function in transmitting paternal epigenetic information between generations.

## Perspectives

Characterizing histone distributions in single spermatozoa will be is key to determine the frequency by which nucleosomes are retained at given genomic locations within a bulk sperm population. Sophisticated functional studies are needed to discern whether and through what mechanisms sperm histones would withstand extensive remodeling of paternal chromatin to contribute to embryonic development and transmission of acquired phenotypes.

## Funding

This project was supported by the Novartis Research Foundation, the 10.13039/501100001711Swiss National Science Foundation (31003A-172873), and the 10.13039/501100000781European Research Council (ERC) under the European Union’s Horizon 2020 research and innovation programme (grant agreement ERC-AdG 695288 — Totipotency).

## CRediT authorship contribution statement

L.G.T. wrote the paper. A.H.F.M.P. supervised L.G.T and edited the paper.

## Conflict of interest statement

Nothing declared.

## Data Availability

No data were used for the research described in the article.

## References

[1] Moritz L., Hammoud S.S. (2022). The art of packaging the sperm genome: molecular and structural basis of the histone-to-protamine exchange. Front Endocrinol.

[2] Balhorn R., Gledhill B.L., Wyrobek A.J. (1977). Mouse sperm chromatin proteins: quantitative isolation and partial characterization. Biochemistry.

[3] Hammoud S.S., Nix D.A., Zhang H., Purwar J., Carrell D.T., Cairns B.R. (2009). Distinctive chromatin in human sperm packages genes for embryo development. Nature.

[4] Brykczynska U., Hisano M., Erkek S., Ramos L., Oakeley E.J., Roloff T.C., Beisel C., Schubeler D., Stadler M.B., Peters A.H. (2010). Repressive and active histone methylation mark distinct promoters in human and mouse spermatozoa. Nat Struct Mol Biol.

[5] Jung Y.H., Sauria M.E.G., Lyu X., Cheema M.S., Ausio J., Taylor J., Corces V.G. (2017). Chromatin states in mouse sperm correlate with embryonic and adult regulatory landscapes. Cell Rep.

[6] Stäubli A., Peters A.H. (2021). Mechanisms of maternal intergenerational epigenetic inheritance. Curr Opin Genet Dev.

[7] Arévalo L., Esther Merges G., Schneider S., Schorle H. (2022). Protamines: lessons learned from mouse models. Reproduction.

[8] Wang T., Gao H., Li W., Liu C. (2019). Essential role of histone replacement and modifications in male fertility. Front Genet.

[9•] Goudarzi A., Zhang D., Huang H., Barral S., Kwon O.K., Qi S., Tang Z., Buchou T., Vitte A.L., He T. (2016). Dynamic competing histone H4 K5K8 acetylation and butyrylation are hallmarks of highly active gene promoters. Mol Cell.

[10] Govin J., Escoffier E., Rousseaux S., Kuhn L., Ferro M., Thévenon J., Catena R., Davidson I., Garin J., Khochbin S. (2007). Pericentric heterochromatin reprogramming by new histone variants during mouse spermiogenesis. J Cell Biol.

[11] De Vries M., Ramos L., Housein Z., De, Boer P. (2012). Chromatin remodelling initiation during human spermiogenesis. Biol Open.

[12] Luense L.J., Wang X., Schon S.B., Weller A.H., Lin Shiao E., Bryant J.M., Bartolomei M.S., Coutifaris C., Garcia B.A., Berger S.L. (2016). Comprehensive analysis of histone post-translational modifications in mouse and human male germ cells. Epigenetics Chromatin.

[13] Bedi Y.S., Roach A.N., Thomas K.N., Mehta N.A., Golding M.C. (2022). Chromatin alterations during the epididymal maturation of mouse sperm refine the paternally inherited epigenome. Epigenetics Chromatin.

[14] Oliva R., Bazett-Jones D., Mezquita C., Dixon G.H. (1987). Factors affecting nucleosome disassembly by protamines in vitro. Histone hyperacetylation and chromatin structure, time dependence, and the size of the sperm nuclear proteins. J Biol Chem.

[15] Dong Y., Isono K.I., Ohbo K., Endo T.A., Ohara O., Maekawa M., Toyama Y., Ito C., Toshimori K., Helin K. (2017). EPC1/TIP60-mediated histone acetylation facilitates spermiogenesis in mice. Mol Cell Biol.

[16] Shiota H., Barral S., Buchou T., Tan M., Couté Y., Charbonnier G., Reynoird N., Boussouar F., Gérard M., Zhu M. (2018). Nut directs p300-dependent, genome-wide H4 hyperacetylation in male germ cells. Cell Rep.

[17] Qian M.X., Pang Y., Liu C.H., Haratake K., Du B.Y., Ji D.Y., Wang G.F., Zhu Q.Q., Song W., Yu Y. (2013). Acetylation-mediated proteasomal degradation of core histones during DNA repair and spermatogenesis. Cell.

[18] Morinière J., Rousseaux S., Steuerwald U., Soler-López M., Curtet S., Vitte A.L., Govin J., Gaucher J., Sadoul K., Hart D.J. (2009). Cooperative binding of two acetylation marks on a histone tail by a single bromodomain. Nature.

[19] Miller T.C., Simon B., Rybin V., Grötsch H., Curtet S., Khochbin S., Carlomagno T., Müller C.W., bromodomain-DNA A. (2016). interaction facilitates acetylation-dependent bivalent nucleosome recognition by the BET protein BRDT. Nat Commun.

[20] Gaucher J., Boussouar F., Montellier E., Curtet S., Buchou T., Bertrand S., Hery P., Jounier S., Depaux A., Vitte A.L. (2012). Bromodomain-dependent stage-specific male genome programming by Brdt. EMBO J.

[21] de la Iglesia A., Jauregi P., Jodar M., Barrachina F., Ded L., Mallofré C., Rodríguez-Carunchio L., Corral J.M., Ballescà J.L., Komrskova K. (2022). H4K5 butyrylation coexist with acetylation during human spermiogenesis and are retained in the mature sperm chromatin. Int J Mol Sci.

[22] Tan M., Luo H., Lee S., Jin F., Yang J.S., Montellier E., Buchou T., Cheng Z., Rousseaux S., Rajagopal N. (2011). Identification of 67 histone marks and histone lysine crotonylation as a new type of histone modification. Cell.

[23] Crespo M., Damont A., Blanco M., Lastrucci E., Kennani S.E., Ialy-Radio C., Khattabi L.E., Terrier S., Louwagie M., Kieffer-Jaquinod S. (2020). Multi-omic analysis of gametogenesis reveals a novel signature at the promoters and distal enhancers of active genes. Nucleic Acids Res.

[24] Luense L.J., Donahue G., Lin-Shiao E., Rangel R., Weller A.H., Bartolomei M.S., Berger S.L. (2019). Gcn5-mediated histone acetylation governs nucleosome dynamics in spermiogenesis. Dev Cell.

[25] Liu S., Yu H., Liu Y., Liu X., Zhang Y., Bu C., Yuan S., Chen Z., Xie G., Li W. (2017). Chromodomain protein CDYL acts as a crotonyl-CoA hydratase to regulate histone crotonylation and spermatogenesis. Mol Cell.

[26] Jha K.N., Tripurani S.K., Johnson G.R. (2017). TSSK6 is required for γH2AX formation and the histone-to-protamine transition during spermiogenesis. J Cell Sci.

[27] Ihara M., Meyer-Ficca M.L., Leu N.A., Rao S., Li F., Gregory B.D., Zalenskaya I.A., Schultz R.M., Meyer R.G. (2014). Paternal poly (ADP-ribose) metabolism modulates retention of inheritable sperm histones and early embryonic gene expression. PLoS Genet.

[28] Meyer-Ficca M.L., Lonchar J.D., Ihara M., Meistrich M.L., Austin C.A., Meyer R.G. (2011). Poly(ADP-ribose) polymerases PARP1 and PARP2 modulate topoisomerase II beta (TOP2B) function during chromatin condensation in mouse spermiogenesis. Biol Reprod.

[29] Lu L.Y., Wu J., Ye L., Gavrilina G.B., Saunders T.L., Yu X. (2010). RNF8-dependent histone modifications regulate nucleosome removal during spermatogenesis. Dev Cell.

[30] Gou L.T., Kang J.Y., Dai P., Wang X., Li F., Zhao S., Zhang M., Hua M.M., Lu Y., Zhu Y. (2017). Ubiquitination-deficient mutations in human Piwi cause male infertility by impairing histone-to-protamine exchange during spermiogenesis. Cell.

[31] Yang G., Chen Y., Wu J., Chen S.H., Liu X., Singh A.K., Yu X. (2020). Poly(ADP-ribosyl)ation mediates early phase histone eviction at DNA lesions. Nucleic Acids Res.

[32] Meyer R.G., Ketchum C.C., Meyer-Ficca M.L. (2017). Heritable sperm chromatin epigenetics: a break to remember. Biol Reprod.

[33] Dottermusch-Heidel C., Gärtner S.M., Tegeder I., Rathke C., Barckmann B., Bartkuhn M., Bhushan S., Steger K., Meinhardt A., Renkawitz-Pohl R. (2014). H3K79 methylation: a new conserved mark that accompanies H4 hyperacetylation prior to histone-to-protamine transition in Drosophila and rat. Biol Open.

[34] Wang X., Kang J.Y., Wei L., Yang X., Sun H., Yang S., Lu L., Yan M., Bai M., Chen Y. (2019). PHF7 is a novel histone H2A E3 ligase prior to histone-to-protamine exchange during spermiogenesis. Development.

[35] Kim C.R., Noda T., Kim H., Kim G., Park S., Na Y., Oura S., Shimada K., Bang I., Ahn J.Y. (2020). PHF7 modulates BRDT stability and histone-to-protamine exchange during spermiogenesis. Cell Rep.

[36] Lafarga V., Sirozh O., Díaz-López I., Galarreta A., Hisaoka M., Zarzuela E., Boskovic J., Jovanovic B., Fernandez-Leiro R., Muñoz J. (2021). Widespread displacement of DNA- and RNA-binding factors underlies toxicity of arginine-rich cell-penetrating peptides. Embo J.

[37] Iuso D., Czernik M., Toschi P., Fidanza A., Zacchini F., Feil R., Curtet S., Buchou T., Shiota H., Khochbin S. (2015). Exogenous expression of human protamine 1 (hPrm1) remodels fibroblast nuclei into spermatid-like structures. Cell Rep.

[38••] Barral S., Morozumi Y., Tanaka H., Montellier E., Govin J., de Dieuleveult M., Charbonnier G., Couté Y., Puthier D., Buchou T. (2017). Histone variant H2A.L.2 guides transition protein-dependent protamine assembly in male germ cells. Mol Cell.

[39] Arévalo L., Merges G.E., Schneider S., Oben F.E., Neumann I.S., Schorle H. (2022). Loss of the cleaved-protamine 2 domain leads to incomplete histone-to-protamine exchange and infertility in mice. PLoS Genet.

[40] Anuar N.D., Kurscheid S., Field M., Zhang L., Rebar E., Gregory P., Buchou T., Bowles J., Koopman P., Tremethick D.J. (2019). Gene editing of the multi-copy H2A.B gene and its importance for fertility. Genome Biol.

[41] Hoghoughi N., Barral S., Curtet S., Chuffart F., Charbonnier G., Puthier D., Buchou T., Rousseaux S., Khochbin S. (2020). RNA-guided genomic localization of H2A.L.2 histone variant. Cells.

[42] Itoh K., Kondoh G., Miyachi H., Sugai M., Kaneko Y., Kitano S., Watanabe H., Maeda R., Imura A., Liu Y. (2019). Dephosphorylation of protamine 2 at serine 56 is crucial for murine sperm maturation in vivo. Sci Signal.

[43••] Gou L.T., Lim D.H., Ma W., Aubol B.E., Hao Y., Wang X., Zhao J., Liang Z., Shao C., Zhang X. (2020). Initiation of parental genome reprogramming in fertilized oocyte by splicing kinase SRPK1-catalyzed protamine phosphorylation. Cell.

[44•] Moritz L., Schon S.B., Rabbani M., Sheng Y., Pendlebury D.F., Agrawal R., Sultan C., Jorgensen K., Zheng X., Diehl A., et al.: **Single residue substitution in protamine 1 disrupts sperm genome packaging and embryonic development in mice**. *bioRxiv* 2021

[45] Bell E.L., Nagamori I., Williams E.O., Del Rosario A.M., Bryson B.D., Watson N., White F.M., Sassone-Corsi P., Guarente L. (2014). SirT1 is required in the male germ cell for differentiation and fecundity in mice. Development.

[46] Pradeepa M.M., Nikhil G., Hari Kishore A., Bharath G.N., Kundu T.K., Rao M.R. (2009). Acetylation of transition protein 2 (TP2) by KAT3B (p300) alters its DNA condensation property and interaction with putative histone chaperone NPM3. J Biol Chem.

[47] Gatewood J.M., Cook G.R., Balhorn R., Bradbury E.M., Schmid C.W. (1987). Sequence-specific packaging of DNA in human sperm chromatin. Science.

[48] Gardiner-Garden M., Ballesteros M., Gordon M., Tam P.P. (1998). Histone- and protamine-DNA association: conservation of different patterns within the beta-globin domain in human sperm. Mol Cell Biol.

[49] Wykes S.M., Krawetz S.A. (2003). The structural organization of sperm chromatin. J Biol Chem.

[50] Arpanahi A., Brinkworth M., Iles D., Krawetz S.A., Paradowska A., Platts A.E., Saida M., Steger K., Tedder P., Miller D. (2009). Endonuclease-sensitive regions of human spermatozoal chromatin are highly enriched in promoter and CTCF binding sequences. Genome Res.

[51] Erkek S., Hisano M., Liang C.Y., Gill M., Murr R., Dieker J., Schubeler D., van der Vlag J., Stadler M.B., Peters A.H. (2013). Molecular determinants of nucleosome retention at CpG-rich sequences in mouse spermatozoa. Nat Struct Mol Biol.

[52] Carone B.R., Hung J.H., Hainer S.J., Chou M.T., Carone D.M., Weng Z., Fazzio T.G., Rando O.J. (2014). High-resolution mapping of chromatin packaging in mouse embryonic stem cells and sperm. Dev Cell.

[53] Samans B., Yang Y., Krebs S., Sarode G.V., Blum H., Reichenbach M., Wolf E., Steger K., Dansranjavin T., Schagdarsurengin U. (2014). Uniformity of nucleosome preservation pattern in Mammalian sperm and its connection to repetitive DNA elements. Dev Cell.

[54••] Yoshida K., Muratani M., Araki H., Miura F., Suzuki T., Dohmae N., Katou Y., Shirahige K., Ito T., Ishii S. (2018). Mapping of histone-binding sites in histone replacement-completed spermatozoa. Nat Commun.

[55••] Yamaguchi K., Hada M., Fukuda Y., Inoue E., Makino Y., Katou Y., Shirahige K., Okada Y. (2018). Re-evaluating the localization of sperm-retained histones revealed the modification-dependent accumulation in specific genome regions. Cell Rep.

[56] Vavouri T., Lehner B. (2011). Chromatin organization in sperm may be the major functional consequence of base composition variation in the human genome. PLoS Genet.

[57] Jung Y.H., Kremsky I., Gold H.B., Rowley M.J., Punyawai K., Buonanotte A., Lyu X., Bixler B.J., Chan A.W.S., Corces V.G. (2019). Maintenance of CTCF- and transcription factor-mediated interactions from the gametes to the early mouse embryo. Mol Cell.

[58] Guo F., Li L., Li J., Wu X., Hu B., Zhu P., Wen L., Tang F. (2017). Single-cell multi-omics sequencing of mouse early embryos and embryonic stem cells. Cell Res.

[59] Li L., Guo F., Gao Y., Ren Y., Yuan P., Yan L., Li R., Lian Y., Li J., Hu B. (2018). Single-cell multi-omics sequencing of human early embryos. Nat Cell Biol.

[60] Chen X., Ke Y., Wu K., Zhao H., Sun Y., Gao L., Liu Z., Zhang J., Tao W., Hou Z. (2019). Key role for CTCF in establishing chromatin structure in human embryos. Nature.

[61] Hernández-Hernández A., Lilienthal I., Fukuda N., Galjart N., Höög C. (2016). CTCF contributes in a critical way to spermatogenesis and male fertility. Sci Rep.

[62] Ke Y., Xu Y., Chen X., Feng S., Liu Z., Sun Y., Yao X., Li F., Zhu W., Gao L. (2017). 3D chromatin structures of mature gametes and structural reprogramming during mammalian embryogenesis. Cell.

[63] Vara C., Paytuví-Gallart A., Cuartero Y., Le Dily F., Garcia F., Salvà-Castro J., Gómez H.L., Julià E., Moutinho C., Aiese Cigliano R. (2019). Three-dimensional genomic structure and cohesin occupancy correlate with transcriptional activity during spermatogenesis. Cell Rep.

[64] Wang Y., Wang H., Zhang Y., Du Z., Si W., Fan S., Qin D., Wang M., Duan Y., Li L. (2019). Reprogramming of meiotic chromatin architecture during spermatogenesis. Mol Cell.

[65] Beagrie R.A., Scialdone A., Schueler M., Kraemer D.C., Chotalia M., Xie S.Q., Barbieri M., de Santiago I., Lavitas L.M., Branco M.R. (2017). Complex multi-enhancer contacts captured by genome architecture mapping. Nature.

[66] Brinkley B.R., Brenner S.L., Hall J.M., Tousson A., Balczon R.D., Valdivia M.M. (1986). Arrangements of kinetochores in mouse cells during meiosis and spermiogenesis. Chromosoma.

[67] Zalensky A.O., Breneman J.W., Zalenskaya I.A., Brinkley B.R., Bradbury E.M. (1993). Organization of centromeres in the decondensed nuclei of mature human sperm. Chromosoma.

[68] Palmer D.K., O'Day K., Margolis R.L. (1990). The centromere specific histone CENP-A is selectively retained in discrete foci in mammalian sperm nuclei. Chromosoma.

[69] Milks K.J., Moree B., Straight A.F. (2009). Dissection of CENP-C-directed centromere and kinetochore assembly. Mol Biol Cell.

[70•] Raychaudhuri N., Dubruille R., Orsi G.A., Bagheri H.C., Loppin B., Lehner C.F. (2012). Transgenerational propagation and quantitative maintenance of paternal centromeres depends on Cid/Cenp-A presence in Drosophila sperm. PLoS Biol.

[71] Palmer D.K., O'Day K., Trong H.L., Charbonneau H., Margolis R.L. (1991). Purification of the centromere-specific protein CENP-A and demonstration that it is a distinctive histone. Proc Natl Acad Sci USA.

[72] Singleton S., Zalensky A., Doncel G.F., Morshedi M., Zalenskaya I.A. (2007). Testis/sperm-specific histone 2B in the sperm of donors and subfertile patients: variability and relation to chromatin packaging. Hum Reprod.

[73] Bodor D.L., Valente L.P., Mata J.F., Black B.E., Jansen L.E. (2013). Assembly in G1 phase and long-term stability are unique intrinsic features of CENP-A nucleosomes. Mol Biol Cell.

[74] Smoak E.M., Stein P., Schultz R.M., Lampson M.A., Black B.E. (2016). Long-term retention of cenp-a nucleosomes in mammalian oocytes underpins transgenerational inheritance of centromere identity. Curr Biol.

[75] Mitra S., Srinivasan B., Jansen L.E.T. (2020). Stable inheritance of CENP-A chromatin: Inner strength versus dynamic control. J Cell Biol.

[76] Amor D.J., Bentley K., Ryan J., Perry J., Wong L., Slater H., Choo K.H. (2004). Human centromere repositioning "in progress". Proc Natl Acad Sci USA.

[77] Tyler-Smith C., Gimelli G., Giglio S., Floridia G., Pandya A., Terzoli G., Warburton P.E., Earnshaw W.C., Zuffardi O. (1999). Transmission of a fully functional human neocentromere through three generations. Am J Hum Genet.

[78] van de Werken C., van der Heijden G.W., Eleveld C., Teeuwssen M., Albert M., Baarends W.M., Laven J.S., Peters A.H., Baart E.B. (2014). Paternal heterochromatin formation in human embryos is H3K9/HP1 directed and primed by sperm-derived histone modifications. Nat Commun.

[79] van der Heijden G.W., Derijck A.A., Ramos L., Giele M., van der Vlag J., de Boer P. (2006). Transmission of modified nucleosomes from the mouse male germline to the zygote and subsequent remodeling of paternal chromatin. Dev Biol.

[80] Meyer-Ficca M.L., Lonchar J.D., Ihara M., Bader J.J., Meyer R.G. (2013). Alteration of poly(ADP-ribose) metabolism affects murine sperm nuclear architecture by impairing pericentric heterochromatin condensation. Chromosoma.

[81] Molaro A., Hodges E., Fang F., Song Q., McCombie W.R., Hannon G.J., Smith A.D. (2011). Sperm methylation profiles reveal features of epigenetic inheritance and evolution in primates. Cell.

[82] Yamagata K., Yamazaki T., Miki H., Ogonuki N., Inoue K., Ogura A., Baba T., Centromeric D.N.A. (2007). hypomethylation as an epigenetic signature discriminates between germ and somatic cell lineages. Dev Biol.

[83] Puschendorf M., Terranova R., Boutsma E., Mao X., Isono K., Brykczynska U., Kolb C., Otte A.P., Koseki H., Orkin S.H. (2008). PRC1 and Suv39h specify parental asymmetry at constitutive heterochromatin in early mouse embryos. Nat Genet.

[84] Tardat M., Albert M., Kunzmann R., Liu Z., Kaustov L., Thierry R., Duan S., Brykczynska U., Arrowsmith C.H., Peters A.H. (2015). Cbx2 targets PRC1 to constitutive heterochromatin in mouse zygotes in a parent-of-origin-dependent manner. Mol Cell.

[85] Zalenskaya I.A., Bradbury E.M., Zalensky A.O. (2000). Chromatin structure of telomere domain in human sperm. Biochem Biophys Res Commun.

[86] Royo H., Stadler M.B., Peters A. (2016). Alternative computational analysis shows no evidence for nucleosome enrichment at repetitive sequences in mammalian spermatozoa. Dev Cell.

[87] Ozturk N., Dansranjavin T., Gies S., Calay D., Shiplu S., Creppe C., Hendrickx J., Schagdarsurengin U. (2021). H4K20me3 marks distal intergenic and repetitive regions in human mature spermatozoa. Development.

[88] Lambrot R., Chan D., Shao X., Aarabi M., Kwan T., Bourque G., Moskovtsev S., Librach C., Trasler J., Dumeaux V. (2021). Whole-genome sequencing of H3K4me3 and DNA methylation in human sperm reveals regions of overlap linked to fertility and development. Cell Rep.

[89] Lu J.Y., Shao W., Chang L., Yin Y., Li T., Zhang H., Hong Y., Percharde M., Guo L., Wu Z. (2020). Genomic repeats categorize genes with distinct functions for orchestrated regulation. Cell Rep.

[90] La Spina F.A., Romanato M., Brugo-Olmedo S., De Vincentiis S., Julianelli V., Rivera R.M., Buffone M.G. (2014). Heterogeneous distribution of histone methylation in mature human sperm. J Assist Reprod Genet.

[91] Ramos L., van der Heijden G.W., Derijck A., Berden J.H., Kremer J.A., van der Vlag J., de Boer P. (2008). Incomplete nuclear transformation of human spermatozoa in oligo-astheno-teratospermia: characterization by indirect immunofluorescence of chromatin and thiol status. Hum Reprod.

[92] Zhang X., San Gabriel M., Zini A. (2006). Sperm nuclear histone to protamine ratio in fertile and infertile men: evidence of heterogeneous subpopulations of spermatozoa in the ejaculate. J Androl.

[93•] Oikawa M., Simeone A., Hormanseder E., Teperek M., Gaggioli V., O'Doherty A., Falk E., Sporniak M., D'Santos C., Franklin V.N.R. (2020). Epigenetic homogeneity in histone methylation underlies sperm programming for embryonic transcription. Nat Commun.

[94] Singleton S., Mudrak O., Morshedi M., Oehninger S., Zalenskaya I., Zalensky A. (2007). Characterisation of a human sperm cell subpopulation marked by the presence of the TSH2B histone. Reprod Fertil Dev.

[95] Štiavnická M., García-Álvarez O., Ulčová-Gallová Z., Sutovsky P., Abril-Parreño L., Dolejšová M., Řimnáčová H., Moravec J., Hošek P., Lošan P. (2020). H3K4me2 accompanies chromatin immaturity in human spermatozoa: an epigenetic marker for sperm quality assessment. Syst Biol Reprod Med.

[96] Gandini L., Lombardo F., Paoli D., Caruso F., Eleuteri P., Leter G., Ciriminna R., Culasso F., Dondero F., Lenzi A. (2004). Full-term pregnancies achieved with ICSI despite high levels of sperm chromatin damage. Hum Reprod.

[97] Niu Z.H., Shi H.J., Zhang H.Q., Zhang A.J., Sun Y.J., Feng Y. (2011). Sperm chromatin structure assay results after swim-up are related only to embryo quality but not to fertilization and pregnancy rates following IVF. Asian J Androl.

[98] Wang C., Chen C., Liu X., Li C., Wu Q., Chen X., Yang L., Kou X., Zhao Y., Wang H. (2022). Dynamic nucleosome organization after fertilization reveals regulatory factors for mouse zygotic genome activation. Cell Res.

[99] van der Heijden G.W., Ramos L., Baart E.B., van den Berg I.M., Derijck A.A., van der Vlag J., Martini E., de Boer P. (2008). Sperm-derived histones contribute to zygotic chromatin in humans. BMC Dev Biol.

[100] Kong Q., Banaszynski L.A., Geng F., Zhang X., Zhang J., Zhang H., O'Neill C.L., Yan P., Liu Z., Shido K. (2018). Histone variant H3.3-mediated chromatin remodeling is essential for paternal genome activation in mouse preimplantation embryos. *J Biol Chem*.

[101] Makino Y., Inoue E., Hada M., Aoshima K., Kitano S., Miyachi H., Okada Y. (2014). Generation of a dual-color reporter mouse line to monitor spermatogenesis in vivo. Front Cell Dev Biol.

[102] Ishiuchi T., Abe S., Inoue K., Yeung W.K.A., Miki Y., Ogura A., Sasaki H. (2021). Reprogramming of the histone H3.3 landscape in the early mouse embryo. Nat Struct Mol Biol.

[103] Wang C., Liu X., Gao Y., Yang L., Li C., Liu W., Chen C., Kou X., Zhao Y., Chen J. (2018). Reprogramming of H3K9me3-dependent heterochromatin during mammalian embryo development. Nat Cell Biol.

[104] Xu Q., Xiang Y., Wang Q., Wang L., Brind'Amour J., Bogutz A.B., Zhang Y., Zhang B., Yu G., Xia W. (2019). SETD2 regulates the maternal epigenome, genomic imprinting and embryonic development. Nat Genet.

[105] Zheng H., Huang B., Zhang B., Xiang Y., Du Z., Xu Q., Li Y., Wang Q., Ma J., Peng X. (2016). Resetting epigenetic memory by reprogramming of histone modifications in mammals. Mol Cell.

[106] Zhang B., Zheng H., Huang B., Li W., Xiang Y., Peng X., Ming J., Wu X., Zhang Y., Xu Q. (2016). Allelic reprogramming of the histone modification H3K4me3 in early mammalian development. Nature.

[107] Lismer A., Dumeaux V., Lafleur C., Lambrot R., Brind'Amour J., Lorincz M.C., Kimmins S. (2021). Histone H3 lysine 4 trimethylation in sperm is transmitted to the embryo and associated with diet-induced phenotypes in the offspring. Dev Cell.

[108] Hammoud S.S., Nix D.A., Hammoud A.O., Gibson M., Cairns B.R., Carrell D.T. (2011). Genome-wide analysis identifies changes in histone retention and epigenetic modifications at developmental and imprinted gene loci in the sperm of infertile men. Hum Reprod.

[109] Schon S.B., Luense L.J., Wang X., Bartolomei M.S., Coutifaris C., Garcia B.A., Berger S.L. (2019). Histone modification signatures in human sperm distinguish clinical abnormalities. J Assist Reprod Genet.

[110] Kimura Y., Yanagimachi R. (1995). Mouse oocytes injected with testicular spermatozoa or round spermatids can develop into normal offspring. Development.

[111] Kurotaki Y.K., Hatanaka Y., Kamimura S., Oikawa M., Inoue H., Ogonuki N., Inoue K., Ogura A. (2015). Impaired active DNA demethylation in zygotes generated by round spermatid injection. Hum Reprod.

[112] Kishigami S., Van Thuan N., Hikichi T., Ohta H., Wakayama S., Mizutani E., Wakayama T. (2006). Epigenetic abnormalities of the mouse paternal zygotic genome associated with microinsemination of round spermatids. Dev Biol.

[113••] Sakamoto M., Ito D., Inoue R., Wakayama S., Kikuchi Y., Yang L., Hayashi E., Emura R., Shiura H., Kohda T. (2022). Paternally inherited H3K27me3 affects chromatin accessibility in mouse embryos produced by round spermatid injection. Development.

[114] Teperek M., Simeone A., Gaggioli V., Miyamoto K., Allen G.E., Erkek S., Kwon T., Marcotte E.M., Zegerman P., Bradshaw C.R. (2016). Sperm is epigenetically programmed to regulate gene transcription in embryos. Genome Res.

[115] Ben Maamar M., Sadler-Riggleman I., Beck D., Skinner M.K. (2018). Epigenetic transgenerational inheritance of altered sperm histone retention sites. Sci Rep.

[116] Bedi Y.S., Wang H., Thomas K.N., Basel A., Prunier J., Robert C., Golding M.C. (2022). Alcohol induced increases in sperm Histone H3 lysine 4 trimethylation correlate with increased placental CTCF occupancy and altered developmental programming. Sci Rep.

[117] Yoshida K., Maekawa T., Ly N.H., Fujita S.I., Muratani M., Ando M., Katou Y., Araki H., Miura F., Shirahige K. (2020). ATF7-dependent epigenetic changes are required for the intergenerational effect of a paternal low-protein diet. Mol Cell.

[118•] Siklenka K., Erkek S., Godmann M., Lambrot R., McGraw S., Lafleur C., Cohen T., Xia J., Suderman M., Hallett M. (2015). Disruption of histone methylation in developing sperm impairs offspring health transgenerationally. Science.

[119] Lismer A., Siklenka K., Lafleur C., Dumeaux V., Kimmins S. (2020). Sperm histone H3 lysine 4 trimethylation is altered in a genetic mouse model of transgenerational epigenetic inheritance. Nucleic Acids Res.

[120] Lambrot R., Xu C., Saint-Phar S., Chountalos G., Cohen T., Paquet M., Suderman M., Hallett M., Kimmins S. (2013). Low paternal dietary folate alters the mouse sperm epigenome and is associated with negative pregnancy outcomes. Nat Commun.

[121] Wu L., Lu Y., Jiao Y., Liu B., Li S., Li Y., Xing F., Chen D., Liu X., Zhao J. (2016). Paternal psychological stress reprograms hepatic gluconeogenesis in offspring. Cell Metab.

[122] Sharma U., Conine C.C., Shea J.M., Boskovic A., Derr A.G., Bing X.Y., Belleannee C., Kucukural A., Serra R.W., Sun F. (2016). Biogenesis and function of tRNA fragments during sperm maturation and fertilization in mammals. Science.

[123] Chen Q., Yan M., Cao Z., Li X., Zhang Y., Shi J., Feng G.H., Peng H., Zhang X., Zhang Y. (2016). Sperm tsRNAs contribute to intergenerational inheritance of an acquired metabolic disorder. Science.

[124] Jung Y.H., Wang H.V., Ruiz D., Bixler B.J., Linsenbaum H., Xiang J.F., Forestier S., Shafik A.M., Jin P., Corces V.G. (2022). Recruitment of CTCF to an Fto enhancer is responsible for transgenerational inheritance of BPA-induced obesity. Proc Natl Acad Sci USA.

[125] Hisano M., Erkek S., Dessus-Babus S., Ramos L., Stadler M.B., Peters A.H. (2013). Genome-wide chromatin analysis in mature mouse and human spermatozoa. Nat Protoc.

[126] Hada M., Masuda K., Yamaguchi K., Shirahige K., Okada Y. (2017). Identification of a variant-specific phosphorylation of TH2A during spermiogenesis. Sci Rep.

[127] Castillo J., Amaral A., Azpiazu R., Vavouri T., Estanyol J.M., Ballescà J.L., Oliva R. (2014). Genomic and proteomic dissection and characterization of the human sperm chromatin. Mol Hum Reprod.

[128] Onikubo T., Nicklay J.J., Xing L., Warren C., Anson B., Wang W.L., Burgos E.S., Ruff S.E., Shabanowitz J., Cheng R.H. (2015). Developmentally regulated post-translational modification of nucleoplasmin controls histone sequestration and deposition. Cell Rep.

[129] Villeponteau B. (1992). Heparin increases chromatin accessibility by binding the trypsin-sensitive basic residues in histones. *Biochem J*.

[130] Chereji R.V., Bryson T.D., Henikoff S. (2019). Quantitative MNase-seq accurately maps nucleosome occupancy levels. Genome Biol.

[131] Saida M., Iles D., Elnefati A., Brinkworth M., Miller D. (2011). Key gene regulatory sequences with distinctive ontological signatures associate with differentially endonuclease-accessible mouse sperm chromatin. Reproduction.

[132] Yin Q., Yang C.-H., Strelkova O.S., Sun Y., Gopalan S., Yang L., Dekker J., Fazzio T.G., Li X.Z., Gibcus J., et al.: **Revisiting chromatin packaging in mouse sperm**. *bioRxiv* 2022. 10.1101/gr.277845.123 PMC10760523 38129076

